# A qualitative study of health care providers’ perceptions and experiences of working together to care for children with medical complexity (CMC)

**DOI:** 10.1186/s12913-018-2857-8

**Published:** 2018-01-31

**Authors:** Lisa Altman, Yvonne Zurynski, Christie Breen, Tim Hoffmann, Susan Woolfenden

**Affiliations:** 1 Sydney Children’s Hospital Network, Sydney, Australia; 2 University of Sydney, Sydney, Australia; 3 University of New South Wales, Sydney, Australia

**Keywords:** Child, Qualitative, Health provider, Children with medical complexity (CMC)

## Abstract

**Background:**

Children with medical complexity (CMC) have a wide range of long term health problems and disabilities that have an adverse impact on their quality of life. They have high levels of family identified health care needs and health care utilisation. There is no Australian literature on the experiences of health care providers working in the Australian tertiary, secondary and primary health care system, whilst managing CMC. This information is essential to inform the design of integrated health care systems for these children. We address this knowledge gap by exploring the perceptions and experiences of health care providers on the provision of health care for CMC aged 0 to 18 years.

**Method:**

A qualitative research study was undertaken. Stakeholder forums, group and individual in depth interviews were undertaken using a semi-structured interview guide. The stakeholder forums were audio recorded and transcribed verbatim. Field notes of the stakeholder forums, group and individual interviews were taken. Inductive thematic analysis was undertaken to identify key themes.

**Results:**

One hundred and three providers took part in the stakeholder forums and interviews across 3 local health districts, a tertiary paediatric hospital network, and primary health care organisations. Providers expressed concern regarding family capacity to negotiate the system, which was impacted by the medical complexity of the children and psychosocial complexity of their families. Lack of health care provider capacity in terms of their skills, time and availability to manage CMC was also a key problem. These issues occurred within a health system that had impaired capacity in terms of fragmentation of care and limited communication among health care providers.

**Conclusion:**

When designing integrated care models for CMC, it is essential to understand and address the challenges experienced by their health care providers. This requires adequate training of providers, additional resources and time for coordination of care, improved systems of communication among services, with timely access to key information for parents and providers.

## Background

Children with medical complexity (CMC) include those with a wide range of chronic physical, developmental and behavioural problems and disabilities that have an adverse impact on their quality of life. At the severe end of this spectrum are those who are medically fragile. This is a heterogeneous and increasingly prevalent group of children with multisystem disease ranging from preterm survivors with cerebral palsy, children with genetic syndromes to adolescent cancer survivors. It is estimated that they comprise only 0.4 to 0.7% of children but they have a high level of family identified health care needs, significant functional limitations and high health care utilisation [1, 2]. These children require highly specialised care, often provided by multidisciplinary teams over their childhood and adolescence [1, 2].

Families of CMC often struggle under the financial, emotional and physical burden of meeting their child’s ongoing needs and navigating a health system that is primarily based on episodic care [3–6]. Their lives are ruled by multiple visits to various medical and non-medical specialists and services that are unlinked and uncoordinated. Families are impacted by time demands, distance travelled, stress, sleep deprivation, comorbid behaviour problems and out of pocket costs [7, 8]. These children are also at greater risk of falling through the gaps of a fragmented and inequitable health care service [9]. This results in poorer health outcomes for the child, unplanned hospital admissions, emergency department (ED) presentations and longer hospital stays, which in turn impacts on their wellbeing [8, 10, 11]. There are also missed opportunities for health promotion and prevention due to a lack of engagement with primary health care services. Fragmented health care is also costly, with uncoordinated care in the USA costing 35% more in health care costs than coordinated care [12].

Integrated health care has been proposed as a way to improve coordination of services for families of CMC internationally and in Australia [13, 14]. The World Health Organisation defines an integrated health service as *“care that is seamless, smooth and easy to navigate”* which *“minimizes both the number of stages in an appointment and the number of separate visits required to a health facility*” [15]. In order to develop a successful integrated health care system one must first understand the experience of parents, CMC and their health care providers. Qualitative research with families who have CMC has identified the need for continuity at all levels of care through better care coordination; improved access to health and social services; high quality communication, and sharing of written medical records and care plans among all health care providers involved in their child’s care [5, 16–18]. Parental empowerment and the quality of the parent-child-health care provider relationship are key in enabling families to navigate the health system successfully [16, 17].

To date, there are few studies internationally that examine the experiences of health care providers who manage CMC. A survey of providers in the United States found that paediatricians were more comfortable with managing CMC than non-paediatricians, however, case management was required to effectively address psychosocial risk in families [19]. Paediatric residents in the US have also identified a lack of care coordination and lack of effective training as key challenges when managing CMC [20].

The Sydney Children’s Hospitals Network (SCHN), a tertiary paediatric hospital network in New South Wales (NSW) Australia, was formed in 2010 bringing together The Children’s Hospital at Westmead and Sydney Children’s Hospital Randwick. The first Clinical Services Plan for SCHN was released in 2012 and one of its key goals is “*closer to home, at home, ambulatory and integrated care”*. It has six key recommendations focused on transforming the experience of patients with CMC and their families. These include: improved care coordination, services closer to home and partnerships for improved care [21]. In 2015, SCHN was successful in securing innovation funding to develop models of integrated care from NSW Health to support families of CMC. Key to designing integrated health care systems for CMC, is the understanding of current perceptions and experiences of health care providers on the provision of health care for CMC. There was no Australian literature available on this topic. We undertook this qualitative research study to address this knowledge gap.

## Methods

### Setting

The project was undertaken across SCHN, which provides over 90% of tertiary paediatric care in NSW with three local health district (LHD) and three primary health network (PHN) partners. SCHN is the largest paediatric health care service in Australia with 86,000 ED presentations, 44,000 inpatient admissions, 136,790 bed days and almost one million outpatient occasions of service each year [22]. In the year from July 2014 to June 2015, 1709 CMC were frequently admitted to SCHN, or frequently attended one of its two EDs [23].

### Recruitment strategy

This project used purposeful sampling of health care providers from a variety of health care services, with variable experience in managing CMC and a broad range of disciplines, backgrounds and job roles [24, 25]. Our partners from the LHDs and PHNs identified providers who had broad experience and were likely to give a variety of insights. Managers in each organisation were also asked by the integrated care project manager (LA) to nominate relevant clinicians. Using a snowballing recruitment technique, these providers were approached by LA and asked to nominate other health care providers to invite to the stakeholder forums, group or individual interviews.

### Ethics, consent and permissions

Ethical and research governance approval for this project was obtained from the Sydney Children’s Hospitals Network Human Research Ethics Committee (HREC Reference: LNR/15/SCHN/299).

The integrated care project manager (LA) contacted potential participants via email, explained the purpose of the forum/interviews and provided them with an information sheet and consent form. Participants returned their signed consent forms before or at the time of the stakeholder forum or interview. Participants were assured of confidentiality and anonymity. Participation was voluntary, and participants were free to withdraw from the study at any time without prejudice. No participants who were approached refused to attend the stakeholder forums or interviews or withdrew their consent.

### Data collection

Data were collected between May and December 2015 at stakeholder forums, group and individual interviews. The stakeholder forums lasted 60–90 min while group and individual interviews lasted 15–45 min. The interviews allowed for detailed exploration of individual and small group experiences while the stakeholder forums allowed expression of a range of views and experiences. Four stakeholder forums were conducted, these were open forums with a minimum of 20 people, across a range of disciplines. Three group interviews were undertaken with several practitioners from the same discipline and local health district, and there were 31 individual interviews. Type of interview was determined by participant preference and logistics. The stakeholder forums were audio-recorded. Group and individual interviews were not audio-recorded, due to participant preference and logistics of the setting, extensive field notes were taken by the project manager instead. No interviews required an interpreter.

Participants in the stakeholder forums, and in all interview settings, were told that the purpose of the integrated care project was to support CMC and their families to navigate the health system. A semi-structured interview guide was used in the stakeholder forums and the interviews. This interview guide was developed based on initial consultations with the integrated project partners. These questions were designed to elicit, context and experiences of challenges, enablers, and perceived needs of families of CMC that were likely to inform the development of a model for integrated care (Table 1). After the first five interviews and first stakeholder forum, this interview guide was modified to include a discussion of emerging themes.

**Table 1:** Interview Guide

Describe your role and the patients in your care? (Asked in individual and group interviews not forum).
Tell me about the children in your care with chronic and complex conditions? E.g. characteristics that help to identify in the children who present most frequently for care, or in the type of care they require?
What problems do these children and their families experience when accessing appropriate care? What does this mean for them?
What services do these children need? Locally, tertiary? Gaps/duplications in the available services?
What care are these children accessing through the tertiary system that could be safely provided locally? What needs to change to provide that care locally?
What care can you provide for these children in your practice/hospital? What support do you need to provide that support?
What have children and their families said they would like to see changed in the delivery of health services? What change would you like to see?

### Data analysis

Stakeholder forum audio-recordings were transcribed verbatim. Audio-recordings were reviewed against the transcripts by the project manager (LA). Field notes of group and individual interviews were also reviewed by LA. As data was collected, thematic analysis was undertaken in an iterative process where the project manager (LA) searched for commonly expressed behaviours, feelings or words. From this initial inductive analysis, themes began to emerge such as ‘complexity’, ‘GP left out of the loop’, ‘communication’ and ‘parental capacity’. Summaries and initial themes of the stakeholder forums, and all interviews were shared with participants for their feedback by the project manager (LA). Participants were asked to comment on the findings and particularly on any areas that they felt had been misunderstood. They were also encouraged to make further comments.

The validity and reliability of the theme development was evaluated using feedback to participants and other stakeholders, and using secondary coders [25]. The emerging themes were also presented to a number of health care providers and consumer groups interested in integrated care who had not been interviewed or taken part in the forums. This generated useful feedback which in turn aided thematic analysis. Secondary coding was undertaken by SW (a paediatrician) and YZ (a health services researcher) neither of whom were interviewed or attended the stakeholder forums. SW and YZ read and coded transcripts independently to identify emergent themes relating to the experience and perceptions of health care providers in managing CMC. LA, SW and YZ then compared and discussed their coding to reach consensus around the final key themes. Data saturation was achieved by all the coders with the following key themes emerging: family capacity, health care provider capacity, system capacity and communication. For each of these key themes the negative case was also elicited [24, 25]. To assist with thematic analysis, data were coded with NVivo 10 software [26].

## Results

### Characteristics of participants

A total of 103 individual stakeholders took part across the three LHDs, three PHNs and the tertiary paediatric network, with strong representation from all disciplines as outlined in Table 2. Sixty-one health care providers took part in 4 stakeholder forums across NSW - 6 allied health providers, 2 community health providers, 17 executive managers/heads of department/directors, 15 GPs, 10 paediatricians/subspecialists, and 11 nurses. Eleven health care providers took part in group interviews (6 nurses, 2 paediatricians, and 3 radiologists) and there were 31 health care providers who had individual interviews (4 allied health providers, 5 executive managers/ heads of department/ directors, 4 GPs, 4 paediatricians, and 14 nurses).

**Table 2:** Characteristics of the Participants

	SCHN	Urban LHD 1	Urban LHD 2	Regional and Rural LHD	Total
Paediatricians	1	7	3	5	16
Sub-Specialists	3	0	0	0	3
Nurses	10	7	7	6	30
Community Health	0	0	3	0	3
Allied Health Practitioners	0	5	1	4	10
General Practice	0	4	3	12	19
Executive/Managers/Directors	2	7	8	5	22
Total	15	30	25	33	103

### Key themes

Key themes to emerge from the thematic analysis included the following challenges in working with CMC:

- Family capacity- to negotiate the system, medical complexity of the children, psychosocial complexity of families.
- Health care provider capacity – their skills, time, availability and resources.
- System capacity –fragmentation of health care services and service culture.
- Communication – among services, health care providers and families.

**Fig. 1:**
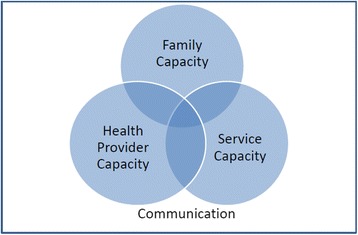
Key Themes

These key themes are represented schematically in Fig. 1.

#### Family capacity (Table 3)

A provider’s ability to manage CMC was impacted by the family’s capacity to coordinate care and navigate the system. This in turn was influenced by the medical complexity of the child, the psychosocial complexity of the family, and parental health literacy.

**Table 3:** Key Themes- Family Capacity

Family Capacity	
Medical complexity	*I think we’re dealing with a very complex heterogeneous group who have got very different needs, and some have got very difficult physical aspects, and some need a lot of behavioural mental health type aspects. (Paediatrician district hospital)* *That baby was born with multiple complications, open heart surgery, he had some lung & kidney problems. After a couple of months, he came home on oxygen and tube feeds. Because of his complex problems he had a number of specialists involved with each. There’s the heart, there’s the lungs, he had a dietician involved and in a hospital, that’s very isolated – one area in the hospital doesn’t talk to another area, but they also don’t think about the impact that it’s having (Community Nurse urban)*
Psychosocial complexity	*Whether the family’s dysfunctional, whether the parent has the illness and the child is being presented as having the problem, but in fact, it’s far more complicated than that. I think complexity can look like all sorts of things. (ED Doctor paediatric hospital)* *A lot of these kids live in remote areas, it adds a whole new level of complexity. (Paediatrician regional)* *Mum said, “I’ve got 2 other children, I’ve just separated from my partner and there’s an AVO, and I’m living on the couches of friends with the kids, relying on them for food. So, at the end of the day, my concern is where I’m going to sleep tonight, not getting my daughter to her appointments”. Even though she’s obese and not worrying that she’s having Coco Pops because at least she’s having something to eat. (GP urban)*
Parental expertise and their health literacy	*I think that having the families carry the knowledge with them, ones that have a background of education do that, they know the condition of the child with a specific syndrome or condition better than 99.9% of the medical staff with those conditions and they carry that information, they have a hard copy to be able to show. I’ve seen it done with hard copies, I’ve seen it done with USB’s, where people leave their USBs at home or hard copy paperwork at home (ED Doctor district hospital)* *The other ones are the hidden ones who…don’t present often, because they’re the families that don’t seek help. They’re the hardest of all because they [see] a range of people, so no one actually sees them. (Paediatric Nurse, district hospital]* *Sometimes their expectation of health and what they want to achieve through it are quite different to what our protocols and policies and our standard of care, our guidelines pathways stipulate. (Paediatrician district hospital)*
Care coordination pressure on the family	*Complexity is all sorts of things and we look at it from a health perspective, but a family’s perspective, you put yourself in those shoes. Navigating health care is really, really complicated. We should be able to make it easier for them. (ED Doctor paediatric hospital)* *Families of children with chronic and complex conditions get tired of telling the story over and over and over again to a new face all the time. (ED Doctor district hospital)* *The challenge that families often face is organising assistance at home. I’ve been involved with a handful here, it’s such a mine field to go through all these different services that are involved (Social Worker district hospital)*

#### Medical complexity

Providers described a wide range of medical complexity for the children in their care, including multisystem conditions which did not fit easily into single disease categories. Such children were often medically fragile with the potential of quickly becoming seriously unwell. They often had a combination of physical and neurodevelopmental disability, feeding difficulties and challenging behavioural and mental health issues. This was compounded by complex equipment needs such as nasogastric tubes and mobility devices, which have maintenance, emergency replacement and supply implications.

#### Psychosocial complexity

Psychosocial complexity was a key determinant perceived to impact on a family’s ability to successfully navigate the system. Many providers described families that were in “chaos”. Financial, cultural and language barriers were key issues impacting on a family’s ability to access services, especially private services. There were transport challenges for children in rural or regional areas including long distances, cost and time. In addition, there was a perception that some disadvantaged groups and those from minority ethnicities were reluctant to attend services due to issues of trust, transport, cost and/or time, regardless of whether they were in urban, regional or rural settings.

#### Parents’ health literacy

Variability of parental health literacy around their child’s condition, and knowledge about which services to access for which medical issue, was another challenge identified. Providers described parents who were not sure when to seek help for their child due to inadequate information about their child’s condition. This sometimes resulted in lengthy delays before bringing children to hospital resulting in worsening of the child’s condition, or bringing children to ED for care that could have been safely accessed in the community. It was felt that the system disempowered families in making decisions about their child’s care and that parents were not involved in discharge planning. However, there were descriptions of families who had a much better understanding of their child’s condition than most health care providers. Many parents had developed methods of carrying their child’s medical information that was helpful when accessing health services, especially unfamiliar health services, or when their child was in crisis.

#### Care coordination pressure on the family

Health care providers felt that there was an increasing expectation by the health care system for parents to coordinate their child’s health care. This was regardless of the family’s capacity, including their level of understanding of their child’s medical complexity, family psychosocial stressors, parental motivation, and availability of money or time. Another identified issue was the mismatch in expectations between parents and health care providers about what the health system could offer. This communication breakdown resulted in families presenting to hospital for a problem that could have been dealt with in the community, had the family received timely advice. In addition, families tired of telling the same story of their children’s complex conditions and medical needs repeatedly. This burden of navigating the health system was felt to have an adverse impact on parental mental health, interactions with siblings, and the CMC.

#### Health provider capacity (Table 4)

The health provider’s capacity in terms of their skill set, time and availability were identified as key factors when managing CMC.

**Table 4:** Key Themes- Health Provider Capacity

Provider capacity	
Skill set	*I know that’s sensitive, but you have to be prepared to do that and link people up proactively, seek a good GP that’s able to do stuff, rather than say, let’s try and make links with your GP when your GP isn’t either interested or able to do it. (Paediatrician district hospital)* *The reason she was in ICU was that the family don’t have a GP, if they have to they go to a medical centre and she describes it as just too difficult, they never know what to do because she’s so complicated, they just see her, and they don’t know what to do. So, she did not get her flu vax, her 2 ½ week stay in ICU was due to influenza type A, completely preventable. She also says that she hadn’t had any of the normal check-ups, she hadn’t had just her basic nutritional bloods done in dot years. There was just all of these little things that had fallen through the cracks for this beautiful young girl (Paediatrician paediatric hospital)* *Also, we should have some sort of education system how to take proper care of the complex, either GP or health professional, there’s some kind of training system. We know the complex problem, but there’s a difficulty in getting the knowledge (Paediatrician district hospital)*
Resources- time availability	*But [even] if you’ve got a good GP, we can’t just spend ½ the day to do a case conference with 2 min’ notice …… and certainly in rural areas, we’re on call and there are days when it just doesn’t work. (GP regional) Many GPs are so busy, they don’t have the time to talk to you and see the information. And again, it’s the situation of where they have a full waiting room and they don’t get paid for the time that they’re spending ……and so sometimes it’s like really hard to put your foot in the door and to be able to help because it’s a shame, because sometimes you can spend a lot of time going around organising case conferencing and then in the end you’re not able to get the GP there. (Community Nurse urban) I always manage to convince the parents to scrimp and save to see the specialist, because 2 years waiting is ridiculous for that child with immense problems with speech to at least have an assessment and get some ideas of how they can help the child. (GP urban)*

#### Skill set

CMC were difficult to manage due to their complexity, even for providers who had training and experience in paediatrics. Because this is a heterogeneous group of children with multiple and sometimes rare conditions, expertise in managing these children required exposure to these conditions. There was a perception that some general practitioners and non-paediatric nurses did not have the skill set to manage CMC. This was compounded by a lack of professional development funding and opportunities. Furthermore, many health care providers, regardless of their paediatric training, struggled when there was a comorbid complex mental health or challenging behaviour component, and/or psychosocial complexity for the family. Providers were prepared to take on complex care if the level of responsibility was well articulated and within their competencies.

There were also issues with providers understanding what services were available to CMC in the broader health care system. Providers as well as parents were described as “lost” in a complex health system. Those in non-tertiary paediatric network services especially struggled with navigating the health system and finding support to manage these children.

#### Resources- time and availability

Time for consultations, time for coordination of care and waiting time for appointments was a key challenge for providers. This time pressure had an adverse impact on communication among services resulting in a lack of opportunities for case conferencing and timely discharge summaries. Managing psychosocial issues and helping families navigate the system was felt to be particularly time costly, but essential. For GPs this was especially challenging because of their practice structure and funding.

Many paediatric services in the LHDs described inadequate funding for current inpatient and outpatient paediatric services, to meet current demand. They described lack of access to medical, nursing, and allied health professionals with paediatric training, diagnostic laboratory, radiology and administrative staff, and paediatric equipment. Another barrier related to difficulties for families and for hospital services to find paediatricians and GPs in the non-hospital sector that provide publicly funded services. Current government funding models were felt to inadequately remunerate time for coordination of care, case management, psychosocial support, allied health therapy and respite.

#### System capacity (Table 5)

The capacity of the system clearly impacted on the effective integrated management of CMC across the primary, secondary and tertiary health care sectors. Key constraints were fragmentation of care and system culture.

**Table 5:** Key Themes- System Capacity

System Capacity	
Fragmentation of care	*We all hear about multiple plans, you have an education health plan that they do themselves, if there’s asthma or diabetes or any medication at your school. FACs have a plan, it’s not just a plan, it’s a health plan. Everyone at the damn table has a plan. Imagine for that one child, it’s not just 2 or 3 or 4, it’s 10 or 11 or 12 plans and what kid or parent is going to pay attention to that. (GP regional)* *There are many different medical teams involved, but each team is focusing on their little bit of the body, and no one is actually taking a broad look at the whole child and the whole family. Particularly complex families get very lost in the middle of that system. (Executive Manager, urban)* *If you want them to see the paediatric gastroenterologist because their inflammatory bowel disease has flared up, why do you need to go and see triage and see a junior doctor in emergency, and see a more senior doctor in emergency and a junior doctor on the gastro team and eventually contact the consultant who knows the patient well to advise how to treat the medication? (ED Doctor paediatric hospital)*
System culture	*The nursing staff were happy to do it but, the ENT department put a block to it and said they don’t want any child with a trachy on the ward ‘cause they’ll ultimately be responsible for it and the irony was that this mother had to care for it herself (Paediatrician district hospital)* *I think it’s dependent on the specialist from each tertiary hospital. Once they’ve had experience with (the local health district) paediatric ward or outpatients or whatever, they then trust you, they get to know you, the care that you’re created. And it is growing, it’s slow but it’s growing. (Paediatric Nurse district hospital)* *In our kids’ ward, if a patient’s there for more than 5 days, parents start worrying that we’re not doing things right. It’s almost a universal base hospital thing, there been there 5 days, they need to go somewhere else. (Paediatrician regional)*

#### Fragmentation of care

Children with multisystem conditions see many subspecialty teams and were particularly vulnerable to fragmentation of the health system and to “falling through the cracks”. Providers commonly described communication difficulties, with a lack of clarity about who is responsible for communicating investigation findings and management plans with anyone other than the family. Adolescents with chronic and complex conditions were particularly vulnerable to service fragmentation when transitioning into adult services. Fragmentation occurred among health services and between health and non-health services such as schools and non-government organisations. Providers talked about partnerships developed at the executive level, but this did not correspond with changes at the service level. Often the only counter measure against fragmentation at a health service level was a passionate individual paediatrician or other health care provider who “held” the child and was their advocate.

Fragmentation of the system meant that for many CMCs the EDs of the tertiary paediatric network and the LHDs were the first point of care when there was equipment failure or a perceived clinical deterioration rather than non-acute health care settings. There were also concerns that many parents were bypassing their GPs, or LHDs, to attend the tertiary paediatric network hospitals.

#### System culture

The culture of the system also acts as a significant barrier to integrated care for CMC. Pathways to health care services were unnecessarily complex and what services offered did not reflect what CMC and their families needed. A lack of clear role delineation within and between the services added to the complexity with duplication of services and gaps. System inertia hampered individual providers when trying to provide care to CMC. For example, some providers described a lack of support from their administrations for the resources required to manage CMC. However, there were also examples where the child’s local care was very well supported by effective informal or ad-hoc collaboration among providers at the tertiary, secondary and primary health care levels.

#### Communication (Table 6)

Health care providers identified limited communication and flow of information across health service boundaries, and to/from families, as a major roadblock in managing CMC.

**Table 6:** Key Themes- Communication

Communication	
Primary Health Care left out of communication	*Sutherland forum* *I’m made to look foolish, scrambling for information. (GP, urban)* *7 out of 10 kids that we see don’t relate to a GP. It’s a bigger issue. And you’re right, we see a lot without GPs. I think one of the issues with complex kids is working out who does what (Paediatrician regional setting)* *So, this is often what happens when the parents present with the child, might be a simple problem, but the background of complex problem, what we probably face is that child might need a simple prescription, but I don’t want to write that not knowing what is going on with the child (GP urban)*
Communication from the tertiary paediatric network	*ED will often say to me that they’ll transfer a child and they’ll never ever know what happens to that child whether it survived, if it’s a resus, or trauma. There’s no feedback from the tertiary facility as to how that child went and they find that quite frustrating at times. (Paediatric Nurse district hospital)* *We need to know the diagnosis. That’s critical, but it’s not always clear in the communication that we get. I think timely communication is probably key. I get letters [from tertiary hospital] that are probably 3 months after they have seen the specialist. To know what actually happens in a timely way. I don’t know why there’s such a gap. By the time you get the letter, the meds have changed 3 times anyway. (GP urban)* *We need to have access to the children’s medical records and pathology from the (tertiary paediatric network). Because sometimes a simple thyroid function test we can access, we can save ½ hour prior to doing all those things. The GP can see it, we can see it from other LHDs, our registrars don’t have to keep ringing everybody. (Paediatrician district hospital)*

#### Primary health care left out of communication

Primary health care practitioners, in particular GPs, reported being left out of communication. They described a lack of understanding about their potential role in co-managing CMC. GPs reported that they were not being sent copies of discharge summaries and outpatient letters and some described their authority to request these being challenged when they contacted the tertiary paediatric network. If parents were given a copy of the letter to give to the GP, the letter was not always brought by parents to their next GP consultation.

Providers from the LHDs and the tertiary paediatric network reported that some families do not have a regular GP due to the difficult psychosocial circumstances for the families, and/or lack of understanding among families of the importance of a regular GP. Community based child and family health nurses also described a lack of awareness of their role resulting in missed opportunities for the universal home visit and monitoring of growth and development. In regional areas where there are fewer paediatric services, GPs played a much more active role in the management of CMC, but this could still be optimised.

#### Communication from the tertiary paediatric network

Delays in obtaining responses to queries from the tertiary paediatric network was frequently described by non-tertiary health care providers. The timeliness of discharge summaries, multiple out of date care plans, and/or overly long care plans were also a key issue. In addition, accessing pathology results for their shared patients from the tertiary paediatric network was cited by many non-tertiary providers as a significant barrier to the effective integrated management of CMC. On the other hand, providers from the tertiary paediatric network described a lack of clarity on what information the referrers required, who to feedback to and which mode of communication to use. The examples of effective communication between the tertiary and non-tertiary sector were mostly reliant on personal relationships between providers rather than systems.

It was clearly articulated that there was a need for a central repository of information rather than siloed information in the multiple health care settings that CMC might attend. Case conferencing was a method suggested to increase communication, but the logistics and resources required were not always available to many providers. Telehealth was also a method that had been used with some success but was not routinely offered.

## Discussion

This qualitative study has explored the key perceptions and experiences of health care providers in providing health care for CMC aged 0 to18 years. It adds essential insights to support health care system changes to provide the best care to CMC.

The issues identified by health care providers in this study regarding the need to enhance family capacity, improve communication, and address system fragmentation have previously been identified in the published qualitative literature as important to families of CMC [5, 16, 17]. Given the high concordance of views between parents and health care providers on these issues, improving these areas has high potential to improve integration of the health care system.

Our study highlights that key to enhancing family capacity is the recognition that the complexity refers not only to medical complexity but also to psychosocial complexity. Research in the USA has demonstrated that families of CMC experience significant financial hardship and inequities in accessing the health care services that they need [6, 9]. When designing integrated health care systems, providers must work in partnership with parents to obtain a comprehensive assessment of the child’s broader ecological context in addition to their complex medical history [6, 9]. This is essential to ensure that the “inverse care law” does not come into play, where the more disadvantaged the family of a CMC, the more adverse the CMC’s health outcomes and the less likely they are to receive services [27]. A comprehensive integrated care program has the potential to act as a “buffer” to address inequities in health care for CMC with greater service provision according to medical and psychosocial need [9, 28].

In addition to supporting family capacity there is a clear need to enhance providers’ capacity to manage CMC. Providers, including paediatricians, described medical training that failed to prepare them to manage this challenging group of children. This is consistent with findings in the US where paediatric trainees felt that they needed training on shared decision making with families of CMC with a clear understanding of psychosocial needs [19, 20]. A framework based on the International Classification of Function, Disability and Health has been proposed to guide training of providers in the care of complex chronic conditions [1]. The Boston Children’s Hospital has also developed a training curriculum for care coordination to equip health care providers with the skills to support families caring for CMC [29]. A recent randomised controlled trial of a web-based multimedia curriculum training paediatric residents from North America in the management of medical complications for CMC found higher levels of satisfaction, improved knowledge and behaviour changes in those residents who received the intervention [30]. A recent publication regarding paediatricians’ understanding and experience with rare diseases indicates that similar programmes are needed in Australia to increase health care providers’ skills and confidence to manage CMC [31].

Several publications have highlighted the importance of the quality of the parent-child-health care provider relationship in managing CMC [16–18]. Key to this is trust and an acknowledgement of shared expertise between families and their health care providers. In our study, providers described instances where trust was at risk due to the fragmentation of services and a lack of clear roles and responsibilities in who does what. Literature on care coordination and the medical home for the child has highlighted that establishing clear roles between families and providers and among different providers is vital to enhance relationships, improve communication and ensure continuity of care [5, 32, 33]. This is especially the case for GPs who described “being left out of the loop” in our study. A lack of engagement with a GP means that well child checks, health promotion opportunities, immunisation and growth monitoring are less likely to occur and that health care for CMC is reactive rather than proactive. It means that CMC may present to hospital ED with mild intercurrent illness rather than seeing their GP. It means that no health care provider is considering the family’s context and health needs overall. Finally, when these children transition to adult services there is no GP who is well acquainted with the whole child and their needs over their life course.

Effective communication between the tertiary and non-tertiary sector in this study was often reliant on personal relationships among providers rather than on systems. The problem with this approach is the risk of communication breakdown when providers change teams or employers. It was clearly articulated that there was a need for a central repository of information, and integrated care plans to move away from siloed information in the multiple health care settings that CMC might attend. [18]This is not only essential for families of CMC but also for their health providers to help them optimise care for the child. All health care providers, carers and CMC need to be included in the development of this central repository to ensure easy access and to optimise its use.

Problems associated with current service fragmentation supports the importance of building up a network of providers and services with experience in managing CMC. There is evidence from research in the USA and Canada that building partnerships across the health system using models of integrated care enable seamless, effective and efficient care that reflects the whole of a CMC’s health needs [13, 34, 35]. Funding models in our health care system need to support this, and to recognise the value of improving service navigation and care coordination for the child, family, health care providers and systems. Funding models based only on activity such as bed days, presentations to health services, number of children seen, are inadequate for this group of children. Models need to shift their focus to health outcomes, quality of life and satisfaction among families, CMC and health care providers.

### Strengths and limitations

A strength of this paper was the systematic approach used in sampling, data collection and analysis to enhance the reliability and validity of the analysis: checking of transcripts against audio-recordings and field notes taken, triangulation among coders by consensus, and feedback of themes to participants, to ensure rigour. In addition, purposeful sampling was used to select a wide range of providers with different experiences [24, 25].

A potential limitation was that although stakeholder forums were audio-recorded and then transcribed, the group and individual interviews were only recorded as field notes by the project manager. Different perceptions and detailed nuances might have come to light if audio-recordings were available for the group and individual interviews.

## Conclusions

This study adds considerably to the understanding of the work we need to do with families, health care providers, and the health care system to ensure that we have effective integrated care models for CMC. An integrated care approach for these children will enable child and family centred care across the health care spectrum, improved communication, bringing care closer to the home and community whenever possible and empowering patients and families to manage their care journey. To do this it is essential that we develop a model where there is a holistic assessment of CMCs needs with clear roles and responsibilities understood and undertaken by all providers involved and their family with respectful sharing of expertise. This requires adequate training of providers, additional resources and time for coordination of care, integration of health care systems and improved timely communication between parents and all providers supporting the CMC. These findings have informed the development and future direction of the SCHN Integrated Care Program.

## Abbreviations

CMC: Children with Medical Complexity
ED: Emergency Department
GP: General Practitioner
HREC: Human Research Ethics Committee
LHD: Local Health District
LNR: Low and Negligible Risk
NSW: New South Wales
PHN: Primary Healthcare Network
SCHN: Sydney Children’s Hospital Network
US: United States of America
